# Removal of Foodborne Pathogen Biofilms by Acidic Electrolyzed Water

**DOI:** 10.3389/fmicb.2017.00988

**Published:** 2017-06-07

**Authors:** Qiao Han, Xueying Song, Zhaohuan Zhang, Jiaojiao Fu, Xu Wang, Pradeep K. Malakar, Haiquan Liu, Yingjie Pan, Yong Zhao

**Affiliations:** ^1^College of Food Science and Technology, Shanghai Ocean UniversityShanghai, China; ^2^Laboratory of Quality and Safety Risk Assessment for Aquatic Products on Storage and Preservation, Ministry of AgricultureShanghai, China; ^3^Shanghai Engineering Research Center of Aquatic-Product Processing and PreservationShanghai, China; ^4^Engineering Research Center of Food Thermal-processing Technology, Shanghai Ocean UniversityShanghai, China

**Keywords:** biofilm, AEW, eradication, EPS, foodborne pathogens

## Abstract

Biofilms, which are complex microbial communities embedded in the protective extracellular polymeric substances (EPS), are difficult to remove in food production facilities. In this study, the use of acidic electrolyzed water (AEW) to remove foodborne pathogen biofilms was evaluated. We used a green fluorescent protein-tagged *Escherichia coli* for monitoring the efficiency of AEW for removing biofilms, where under the optimal treatment conditions, the fluorescent signal of cells in the biofilm disappeared rapidly and the population of biofilm cells was reduced by more than 67%. Additionally, AEW triggered EPS disruption, as indicated by the deformation of the carbohydrate C-O-C bond and deformation of the aromatic rings in the amino acids tyrosine and phenylalanine. These deformations were identified by EPS chemical analysis and Raman spectroscopic analysis. Scanning electron microscopy (SEM) images confirmed that the breakup and detachment of biofilm were enhanced after AEW treatment. Further, AEW also eradicated biofilms formed by both Gram-negative bacteria (*Vibrio parahaemolyticus*) and Gram-positive bacteria (*Listeria monocytogenes*) and was observed to inactivate the detached cells which are a potential source of secondary pollution. This study demonstrates that AEW could be a reliable foodborne pathogen biofilm disrupter and an eco-friendly alternative to sanitizers traditionally used in the food industry.

## Introduction

Foodborne pathogens which persist in food processing facilities grow predominantly as biofilms rather than in planktonic mode (Barnes et al., 1999; Bae et al., 2012). Biofilms are complex communities of microorganisms attached to biotic or abiotic surfaces and protected them by providing firm three-dimensional, multicellular, complex, self-assembled structures that contain extracellular polymeric substances (EPS) (exopolysaccharides, proteins, and extracellular DNA, etc.) (Costerton et al., 1999; Hall-Stoodley et al., 2004). More than 80% of the bacterial infections in the human population are associated with biofilms and approximately 60% of foodborne outbreaks are caused by biofilms (Wolcott and Ehrlich, 2008; Simoes et al., 2010; Bridier et al., 2015). Furthermore, compared to planktonic cells, biofilm-associated cells are more resistant to external stresses such as antibiotics and detergents, thus they are extremely difficult to eliminate resulting in the onset of foodborne illness (Costerton et al., 1987; Hoiby et al., 2010).

It is well documented that biofilm development is one of the most complex physiological processes. Importantly, in the dynamic process, EPS facilitates the trapping of nutrients and maintenance of the structure integrity of the biofilm while also providing a sanctuary for the encased bacterial cells (Li and Yu, 2011; Bassin et al., 2012). Pathogens encased in the EPS-rich matrix, therefore provide a source of contamination when the biofilm interacts with food materials (Carpentier and Cerf, 1993). In addition, the structure of EPS reduces disinfectants access and possibly triggers bacterial tolerance to commonly used sanitizers (Flemming and Wingender, 2010; Xiao et al., 2012; Koo et al., 2013; Lebeaux et al., 2014).

The mode of action of conventional sanitizers to control of foodborne pathogen biofilms is antibacterial rather than EPS matrix disruption (Gao et al., 2016), and any bacteria in the biofilms that survives the sanitizer treatment may initiate biofilm regrowth (Bridier et al., 2015). Thus, novel approaches that include disruption of EPS formation and at the same time kills or removes biofilm cells would be highly desirable (Allaker and Memarzadeh, 2014; Wang et al., 2016). A recent candidate is acidic electrolyzed water (AEW) which has attracted attention in recent years as a promising sanitizing agent in the food, medical, and agricultural industries (Wang et al., 2014). AEW is generated by anodic electrolysis of dilute NaCl solutions and the physicochemical properties include low pH, available chlorine concentration (ACC) and oxidation reduction potential (ORP) (Kim et al., 2000; Xiong et al., 2010; Hao et al., 2012).

Acidic electrolyzed water has been documented to be an effective disinfectant for inactivating foodborne pathogens including *Escherichia coli, Vibrio parahaemolyticus*, and *Listeria monocytogenes* (Kim et al., 2000; Wang et al., 2014). The postulated mode of action is reduction of cell wall, nucleus, and outer membrane integrity which leads to the rapid leakage of intracellular DNA and proteins (Zeng et al., 2010, 2011; Ding et al., 2016). Additionally, AEW is an environmental friendly sanitizer and poses minimal risk to human health (Mori et al., 1997; Wang et al., 2014).

Above all, many studies have shown the bactericidal effect of AEW on planktonic pathogens, but study on the applying the AEW for removing foodborne pathogen biofilms is still lacking. Therefore, this study attempted to use the AEW as a novel scavenger to control foodborne pathogen biofilms, and evaluated the eradication effect of AEW on biofilms and EPS disruption.

## Materials and Methods

### Bacterial Strain and Culture Preparation

*Escherichia coli* K-12 strain ATCC 25404 was used as a model biofilm-forming strain. To generate a fluorescent variant, *E. coli* was transformed with the GFP plasmid pCM18 (Hansen et al., 2001), which conferred resistance to erythromycin. *E. coli* were grown overnight in Luria Bertani (LB, Land Bridge Technology, Beijing, China) broth containing 100 μg/mL ampicillin and IPTG with shaking (250 rpm) at 37°C. *V. parahaemolyticus* S36 and *L. monocytogenes* WaX12 used this study were isolated and stored in our laboratory. *V. parahaemolyticus* S36 and *L. monocytogenes* WaX12 were isolated from shrimp and pork samples by using specific selective media, species-specific gene and API system tests (BioMérieux, Marcyl’Etoile, France). *V. parahaemolyticus* S36 was cultured in tryptic soy broth (TSB, Beijing Land Bridge Technology Company Ltd, Beijing, China) plus 3% NaCl. *L. monocytogenes* WaX12 serotype 1/2a was grown in brain heart infusion (BHI, Land Bridge Technology, Beijing, China). The cultures were diluted to obtain a bacteria population of 9 log CFU/mL.

### Biofilms Formation

Biofilm formation experiments were carried out as described previously (Krom and Willems, 2016; Song et al., 2016) with minor modifications. Static biofilms were grown in 24 well polystyrene microtiter plates (Sangon Biotech Co., Ltd, Shanghai, China). The test bacteria cultures were diluted in fresh culture medium (1:100) and aliquoted into wells. *E. coli* was incubated statically to form biofilms for various time (2, 4, 6, 8, 10, 12, 24, and 48 h). *L. monocytogenes* and *V. parahaemolyticus* were incubated statically to form biofilms for 48 h (Ayebah et al., 2006; Song et al., 2016).

### Preparation of Acidic Electrolyzed Water

Acidic electrolyzed water was prepared according to Wang et al. (2014). The AEW generator model FW-200 (AMANO Corporation, Kanagawa, Japan) was ran for 15 min with the amperage set as 10 A before collection of a sample for testing. The pH and ORP were determined using a pH/ORP meter (model pH 430, Corning Life Sciences, New York, United States). The ACC in AEW was determined by a colorimetric method using a digital chlorine test kit (RC-2Z, Kasahara Chemical Instruments Corp., Saitama, Japan). All measurements were carried out in triplicate. The physicochemical properties of each AEW are shown in **Table 1**.

**Table 1:** Physicochemical properties of AEW electrolyzed by the different NaCl concentration.

NaCl concentration (g/L)	pH	ORP (mV)	ACC (mg/mL)
0.1	2.94 ± 0.05	1087.07 ± 1.85	8.67 ± 0.58
1	2.23 ± 0.01	1172.60 ± 3.47	48.33 ± 2.89
3	2.30 ± 0.03	1175.50 ± 3.84	136.33 ± 2.08
5	2.46 ± 0.05	1173.37 ± 3.93	173.67 ± 1.15

### Crystal Violet Staining Method and MTT Assay

After incubation, biofilm production was quantified using a crystal violet staining method as described previously by Antoniani et al. (2010). Biofilms in the wells of the polystyrene microtiter plates were air-dried for 10 min, then stained with 1 mL of 0.1% (w/v) crystal violet (Sangon Biotech Co., Ltd, Shanghai, China) for 30 min. The wells were then washed three times with 0.1 M phosphate-buffered saline (PBS) (Sangon Biotech Co., Ltd, Shanghai, China). Biofilm was solubilized using 1 mL of 95% ethanol (Sinopharm Chemical Reagent Co., Ltd., Shanghai, China) for 30 min. The optical density of each well was measured at wavelength of 600 nm.

The viability of the biofilm cells was measured by 3-(4,5-dimethylthiazol-2-yl)-2,5-diphenyl tetrazolium bromide (MTT) assay which has been attributed to Mshana et al. (1998) and Krom et al. (2007). One milliliter of culture medium and 0.1 mL of 5 mg/ml MTT solution were added to each well, then incubated at 37°C for 2 h. The culture supernatant was then discarded and 1 mL of dimethyl sulfoxide was added to each well to solubilize the MTT for 2 h. The optical density of each well was measured at wavelength of 570 nm. All measurements were carried out in triplicate and the mean data are presented.

### AEW Treatment on Established Biofilms

Following biofilm formation, the suspension was gently aspirated from the plate and the wells were rinsed three times with PBS to remove non-adherent cells. The biofilms were then exposed to 1 mL sterile deionized water (SDW) or AEW produced by different NaCl concentration (0.1, 1, 3, and 5 g/L) marked AEW-1, AEW-2, AEW-3, AEW-4, respectively, at room temperature (25 ± 1°C). Subsequently, 1 mL neutralizing agent (PBS containing 0.8% Na_2_S_2_O_3_) was added to stop the bactericidal effects of AEW after a 30 s treatment (Luppens et al., 2002; Wang et al., 2014). Surviving cells were collected by vortexing and scraping of the wells and transferred to tubes containing sterile 0.85% NaCl solution. Serially dilution of the bacterial population was plated onto correspondent agar and incubated at 37°C for 24 h. Three replicates were tested for each treatment. According to Wang et al. (2016), the percentage of reduction biofilm cells (%) = (the cells numbers in control group - the cells numbers in treatment group)/the cells numbers in control group × 100. The percentage of reduction biofilm cells represents the removal efficiency.

### Visualization of the Biofilms Using Epifluorescence and Scanning Electron Microscopy

Biofilms which were treated with AEW-3 and SDW and untreated control, were then fixed with 4% glutaraldehyde overnight, and dehydrated in an ascending acetonitrile series (30, 50, 70, 80, 90, and 100% twice for 10 min each). Samples were sputtered with gold and observed with a Nova 450 scanning electron microscope (FEI, Hillsboro, OR, United States). Epifluorescence visualizations were carried out without previous fixation or dehydration and directly observed in a EVOS^®^ FL Auto Cell Imaging System (AMG, Thermo Fisher Scientific, Waltham, MA, United States). We initially observed different areas in one sample at low magnification. Then we choose one area in each sample based on similarity of all images and use high magnification of this area for analysis. The images from three independent experiments with three replications were used for analysis. Pictures were obtained using the same settings for each picture. The fluorescent density in the biofilm cells were quantified by the ImageJ software (National Institutes of Health, Bethesda, MD, United States)^1^.

### EPS Chemical Analysis

Extracellular polymeric substance in a biofilm was extracted using the sonication method (Liu et al., 2007; Gong et al., 2009; D’Abzac et al., 2010). The density of suspended cultures was initially measured at OD_595 nm_. The biofilm cells were then collected by vortexing and scraping in 1 mL 0.01 M KCl solution. The cells were pretreated with a sonicator (VCX 500, SONICS, Newtown, CT, United States) for four cycles of 5 s of operation and 5 s of pause at a power level of 3.5 Hz. The sonicated suspension was centrifuged (4,000 rcf, 20 min, 4°C), and the supernatant was then filtered through a 0.22 μm membrane filter (Sangon Biotech Co., Ltd., Shanghai, China). The amounts of protein and carbohydrate in the filtrate were analyzed. The amounts of carbohydrate and protein were quantified by the phenol–sulfuric acid method and Lowry method (Kim and Park, 2013; Nakamura et al., 2013). The amount of protein and carbohydrate were quantified by OD_750 nm_/OD_595 nm_ and OD_490 nm_/OD_595 nm_, respectively. Each experiment was carried out at least three times.

### Raman Spectroscope

Extracellular polymeric substance was extracted as described in EPS chemical analysis. All Raman spectrum were obtained by a Senterra R200-L Dispersive Raman Microscope (Bruker Optics, Ettlingen, Germany) at room temperature. Raman spectrum of each sample was determined as the average of fifteen measurements at different random sites on the biofilm. The Raman measurements were recorded with an accumulation time of 60 s in the 500–1250 cm^-1^ range. Raman spectral acquisition and preprocessing of preliminary data were carried out using the Bruker OPUS software.

### Statistical Analysis

The percentage of reduction biofilm cells, carbohydrate and protein content in EPS and Raman intensity were analyzed by analysis of one-way ANOVA using the SPSS statistical software (version 19.0; SPSS Inc., Chicago, IL, United States). The level of statistical significance was *p* < 0.05.

## Results

### The Dynamic Development of *E. coli* Biofilm Formation

**FIGURE 1:**
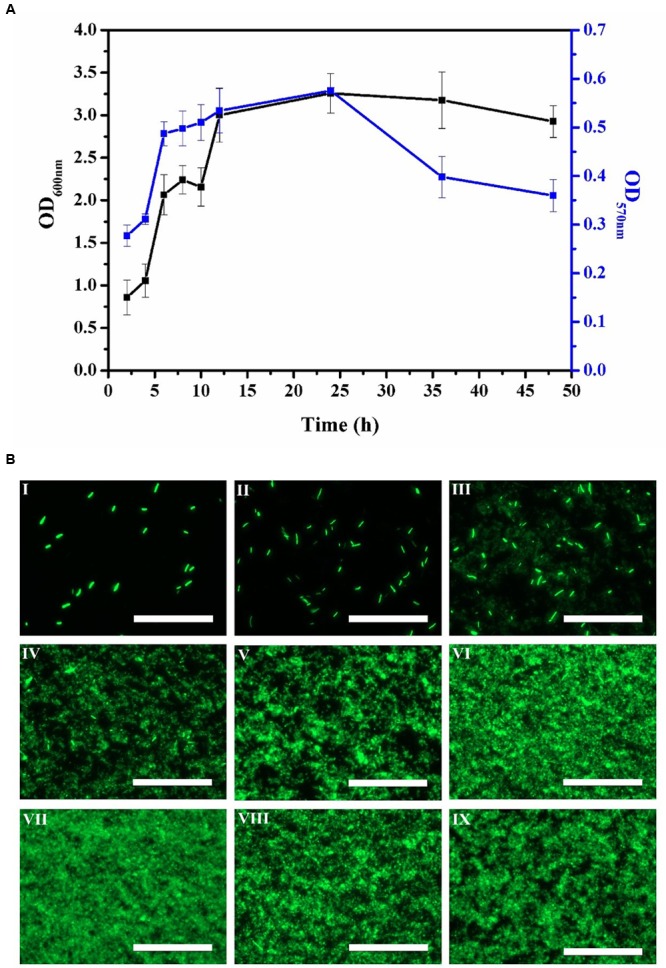
Time course of *E. coli* biofilm production for 2, 4, 6, 8, 10, 12, 24, 36, and 48 h. **(A)** Biofilm biomass (OD_600 nm_) by crystal violet staining method and biofilm viability (OD_570 nm_) by MTT assay. Error bars indicated standard deviations of triplicate experiments. **(B)** Fluorescence images during *E. coli* biofilm development process by epifluorescence microscopy, **I-IX** represented 2, 4, 6, 8, 10, 12, 24, 36, and 48 h, respectively. The scale bar represented 100 μm. Pictures were representative of three independent experiments with three replicates each.

Bacterial biofilm formation is a dynamic process with distinct phases of development (Hall-Stoodley et al., 2004). In principle, there are three stages of biofilm development: an initial attachment and growth phase, a mature phase and a dispersal phase. In our model system, stage one was evident when the number of small homogeneous green-fluorescent colonies of biofilm cells gradually increased (**Figures 1BI–III**). **Figure 1A** shows the corresponding increase in cell viability and biomass of the biofilm from 2 h to 6 h. Stage two of the biofilm developmental process could be seen from 8 h to 24 h where we observed the aggregation of cells into mature biofilm (**Figures 1A,BIV–VII**). The mature biofilm showed green fluorescence exclusively in their border regions, resulting in multilayer films of bacterial cells. This indicated that the biofilm had been established and the biomass within the biofilm was relatively constant. After 24 h of cultivation, stage three commenced and the biomass appeared disaggregated as transient motility of the biofilm cells led to dispersal (**Figures 1BVIII,IX**).

### The Eradication Effect of AEW on *E. coli* Biofilm

**FIGURE 2:**
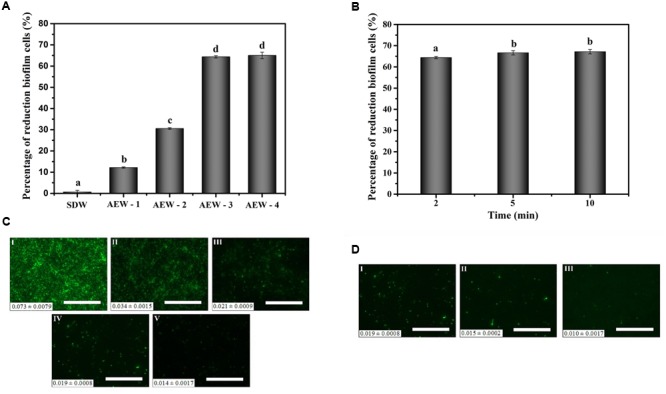
Effect of AEW on *E. coli* cells within biofilms after the SDW, AEW-1, AEW-2, AEW-3, and AEW-4 treatments for different times. Fluorescence density value was presented at the bottom left of each image. The percentages of reduction biofilm cells after the SDW, AEW-1, AEW-2, AEW-3, and AEW-4 treatments for 2 min **(A)**, and the fluorescence images changes of *E. coli* cells within biofilms after the SDW **(C, I)**, AEW-1 **(C, II)**, AEW-2 **(C, III)**, AEW-3 **(C, IV)**, and AEW-4 **(C, V)** treatments for 2 min, respectively. The percentages of reduction biofilm cells after exposed to the AEW-3 treatments for 2, 5, and 10 min **(B)**, and the fluorescence images of *E. coli* cells within biofilm after exposed to the AEW-3 for 2 min **(D, I)**, 5 min **(D, II)**, 10 min **(D, III)**, respectively. The treatment condition of the image **C (IV)** was the same as that for the Image **D (I)**. Scale bar represented 100 μm. Error bars indicated standard deviations of triplicate experiments, and the same letter represented no significant difference (*P* ≥ 0.05).

The numbers of *E. coli* cells in the biofilm after 24 h were approximately 6.77 log CFU/mL as confirmed by the plate count method. **Figure 2A** showed the effect of the different AEW treatments to cell numbers in the mature biofilms in our model system. AEW-1, AEW-2, AEW-3, and AEW-4 were produced using increasing concentrations of NaCl and the reduction in cell number was positively correlated to the NaCl concentration. The bactericidal activity of the AEW-3 treatment was optimal and the trend in reduction of biofilm cell number was confirmed by the visualization of *E. coli* biofilms using epifluorescence microscopy (**Figure 2C**). These images showed that only a few scattered viable cell aggregates were observed in the biofilm after 2 min exposure to AEW. The fluorescent intensity, an indication of cell density and viability, was still at maximum intensity after the SDW treatment and then decreased progressively after the AEW treatments. The fluorescent intensity was minimal after AEW-3 treatment and we selected this treatment for further analysis.

Increasing the contact time with AEW-3 from 2 to 10 min increased cell death and removal, minimally (**Figures 2B,D**). Taken together, it was observed that increasing the potency of AEW using increasing NaCl concentration was more important than treatment time for the eradication of *E. coli* biofilm cells. In addition, the number of viable cells which escaped from the biofilm after AEW-3 treatment was below the limit of detection (<1.4 log CFU/mL). However, the residual viable cells after SDW treatment were as much as 6 log CFU/mL. We chose AEW-3 exposure for 5 min for further experimentation.

### EPS Analysis

**FIGURE 3:**
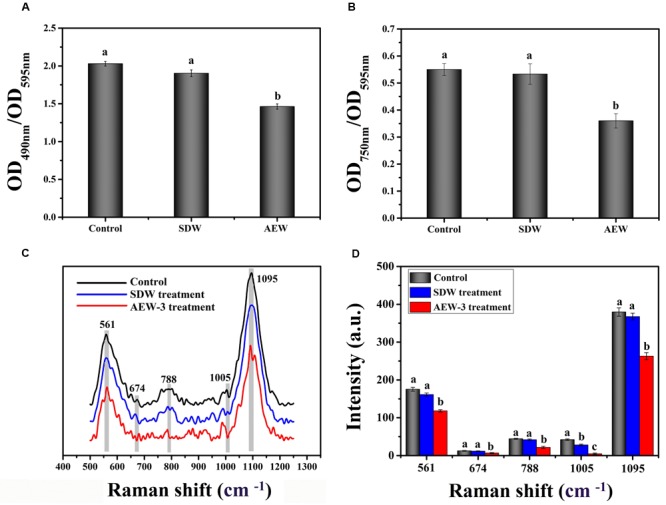
Chemical composition and contents of EPS which represent integrity in *E. coli* biofilms, with untreated, SDW and AEW-3 treatment. **(A)** Total carbohydrates (OD_490 nm_/OD_595 nm_) and **(B)** total protein (OD_750 nm_/OD_595 nm_) in EPS of *E. coli* biofilms. Raman spectrum **(C)** and intensities changes **(D)** of *E. coli* biofilm after untreated, treatment with SDW and AEW-3 for 5 min. Error bars indicated the standard deviations of five measurements and the same letter represented no significant difference (*P* ≥ 0.05).

To test the effect of AEW on EPS production, we analyzed the total carbohydrate and protein content of EPS in a 24 h *E. coli* biofilm. As shown in **Figures 3A,B**, both total carbohydrate and total protein were reduced after exposure to SDW and AEW-3 for 5 min. However, total protein was reduced more than total carbohydrate. The total protein with AEW was 65% of the control, whereas total carbohydrate with AEW was 72% of the control. However, SDW treatment resulted in only little reduction of carbohydrate and protein, and had no significant difference compared to control.

Representative Raman spectra of EPS in the spectral fingerprint range of 500–1250 cm^-1^ are presented in **Figure 3C**. The tentative peak assignments of the bands are summarized in **Table 2**. The prominent Raman bands of EPS belonged to carbohydrates: 561 cm^-1^ (C-O-C glycosidic ring def polysaccharide) and 1090–1095 cm^-1^ (C-O-C glycosidic link). The band intensity weakened dramatically with AEW treatment. Besides, EPS after AEW treated showed a shift in 561 cm^-1^ toward 581 cm^-1^, 1095 cm^-1^ toward 1106 cm^-1^. Two broad Raman bands at 1020–1085 cm^-1^ and 855–899 cm^-1^ were assigned to C-C stretching of carbohydrates (polysaccharides). After AEW-3 treatment, these intensities were significantly weakened as shown in **Figure 3D**.

**Table 2:** Assignment of Raman bands of EPS in biofilms.

Peak position (cm^-1^)	Assignment	Reference
561–582	C-O -C glycosidic ring def polysaccharide; COO^-^ wag; C-C skeletal	Laucks et al., 2005; Guicheteau et al., 2008; Ivleva et al., 2008
637–695	C-S str and C-C twisting of proteins (tyrosine)	Laucks et al., 2005; Guicheteau et al., 2008; Kahraman et al., 2009
782–788	O-P -O str of DNA	Samek et al., 2014
830–850	Tyr	Schwartz et al., 2009
855–899	C-C str, C-O-C 1,4 glycosidic link	Wagner et al., 2009; Ivleva et al., 2009.
1003, 1005	Ring breath Phe	Laucks et al., 2005; Çulha et al., 2008; Guicheteau et al., 2008; Ivleva et al., 2008; Kahraman et al., 2009
1020–1085	C-C , and C-O str (carbohydrates)	Schwartz et al., 2009
1090–1095	C-C str, C-O-C glycosidic link; ring br, sym	Schenzel and Fischer, 2001; Maquelin et al., 2002; De Gussem et al., 2005; Harz et al., 2005; Neugebauer et al., 2007

*def, deformation vibration; str, stretching; Tyr, tyrosine; Phe, phenylalanine; br, breathing; sym, symmetric.*

Extracellular polymeric substance showed a decrease in the magnitude of Raman intensity at 637–695 cm^-1^, 830–850 cm^-1^, 1003 cm^-1^, and 1005 cm^-1^, the bands which corresponds to proteins. For example, bands at 1005 cm^-1^ could be observed in the spectrum of the control group and SDW group. However, these bands were not present in the spectra of EPS after AEW treatment.

The reduction of Raman intensity corresponding to DNA, such as the bands at 788 cm^-1^ and 830–850 cm^-1^, arises from the destruction of the ring structure, indicating degradation of the DNA. This reduction provides further evidence for cell death or removal.

### SEM Analysis

**FIGURE 4:**
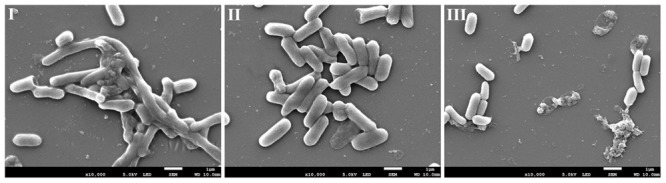
Representative photomicrographs by SEM of biofilm formed by *E. coli* after untreated **(I)**, treated with SDW **(II)**, and AEW-3 for 5 min **(III)**. Scale bar represented 5 μm. Pictures were representative of three independent experiments with three replicates each.

To gain further insight into the mode of action of AEW in eradicating biofilms, SEM images of *E. coli* biofilm treated with AEW were performed. Representative SEM images of *E. coli* biofilm are shown in **Figure 4**. Overall, the untreated samples revealed biofilms had well organized network structures encased in a protective EPS. After SDW treatment, there were still aggregates of cells held together by EPS. However, there were significant differences after AEW treatment, where only a few cells were scattered sporadically and cell lysis was evident when compared to control. The SEM images revealed that most of the biofilm cells were detached from the biofilm matrix and suggested that EPS was disrupted after AEW treatment.

### Effect of AEW on Other Foodborne Pathogen Biofilms

While AEW demonstrated activity against *E. coli* biofilms, it was of interest to explore whether biofilms formed by other foodborne pathogens such as *L. monocytogenes* and *V. parahaemolyticus*, were also sensitive to AEW.

**FIGURE 5:**
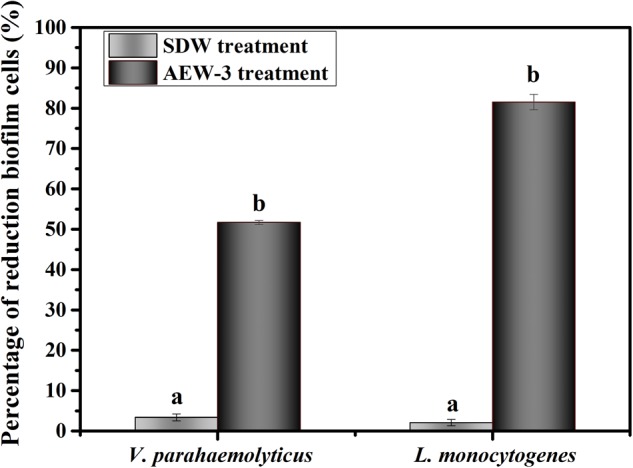
Percentage of reduction biofilm cells of *V. parahaemolyticus* and *L. monocytogenes*.

The efficacy of AEW in eradicating biofilm cells of *L. monocytogenes* and *V. parahaemolyticus* are showed in **Figure 5**. The populations of *L. monocytogenes* and *V. parahaemolyticus* biofilm cells were decreased by 82 and 52% after AEW treatment. There were significant differences obviously between the reduction of biofilm cell number after AEW treatment and those after SDW treatment (*p* < 0.05). SDW treatment marginally reduced the population of biofilm cells of *L. monocytogenes* and *V. parahaemolyticus*. In contrast, the *L. monocytogenes* biofilm was more susceptible to AEW treatment than the *V. parahaemolyticus* biofilm. Additionally, the detachment of residual viable cells from a biofilm after AEW treatment was markedly less when compared to biofilms treated with SDW (data were not shown). After AEW treatment, the detached cells which were a potential source of secondary pollution were lower than detecting value.

**FIGURE 6:**
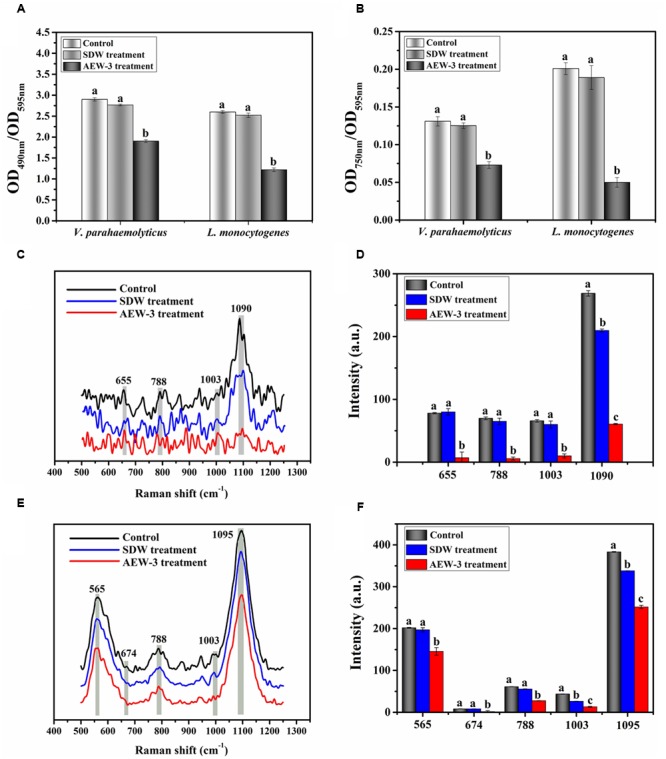
Chemical composition and contents of EPS in *V. parahaemolyticus* and *L. monocytogenes* biofilms with untreated, SDW and AEW-3 treatment. **(A)** Total carbohydrates (OD_490 nm_/OD_595 nm_) and **(B)** total protein (OD_750 nm_/OD_595 nm_) in EPS. Raman spectrum changes of *V. parahaemolyticus* **(C)** and *L. monocytogenes* **(E)** biofilm. Raman intensity changes of *V. parahaemolyticus* **(D)** and *L. monocytogenes* **(F)** biofilm. Error bars indicated the standard deviations of five measurements and the same letter represented no significant difference (*P* ≥ 0.05).

Extracellular polymeric substance analysis of *V. parahaemolyticus* and *L. monocytogenes* biofilm is shown in **Figures 6A,B**. SDW treatment only result in 3–6% EPS reduction and there was no significant difference between SDW treatment and control. The carbohydrate and protein content of EPS in *V. parahaemolyticus* were reduced by 34 and 44%. Comparatively, there was a reduction of 53% carbohydrate and 75% protein content in EPS of *L. monocytogenes* biofilm. AEW treatment therefore is more effective in reducing protein than carbohydrate content in EPS of *L. monocytogenes* and *V. parahaemolyticus*. Representative Raman spectra of EPS in the spectral fingerprint range of 500–1250 cm^-1^ are presented in **Figures 6C–F**. The Raman bands of EPS in *L. monocytogenes* biofilm at 1090 cm^-1^ (C-O-C glycosidic link) were decreased after AEW treatment, indicating the destruction of the carbohydrate structure (**Figures 6C,D**). Similar trends were observed for carbohydrates at band 565 cm^-1^ (C-O-C glycosidic ring def polysaccharide) and band 1095 cm^-1^ (C-O-C glycosidic link).

The band intensity was also weakened by a big margin after AEW treatment as shown in **Figures 6E,F**. Raman intensity in *V. parahaemolyticus* and *L. monocytogenes* biofilm was also reduced at bands 637–695 cm^-1^ and 1003 cm^-1^ (proteins) after AEW treatment. The Raman bands at 1,003 cm^-1^ could be observed in the spectra of the control and SDW group, but these bands were not present in the spectra of EPS in *L. monocytogenes* biofilm after AEW treatment. The decrease of Raman intensity at band 788 cm^-1^ which corresponds to DNA, arises from the destruction of the ring structure and indicated the degradation of DNA.

**FIGURE 7:**
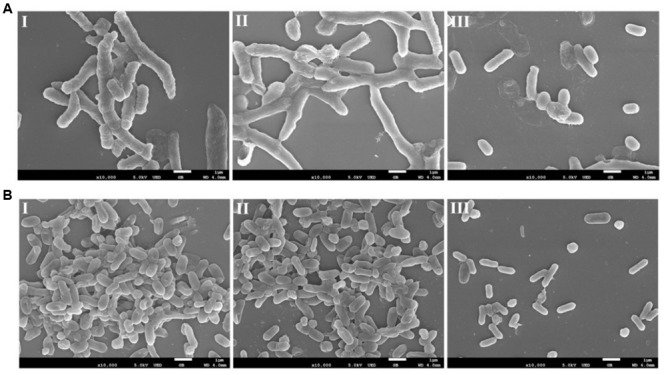
Representative photomicrographs by SEM of biofilm formed by *V. parahaemolyticus* **(A)** and *L. monocytogenes* **(B)** after untreated **(I)**, treated with SDW **(II)**, and AEW-3 for 5 min **(III)**. Scale bar represented 5 μm. Pictures were representative of three independent experiments with three replicates each.

The eradication effect of AEW on biofilm formed by *L. monocytogenes* and *V. parahaemolyticus* was further evaluated using SEM. Representative SEM images (**Figures 7A,B**) show that untreated biofilm had nearly uniform and dense mature architecture. *L. monocytogenes* biofilm forms a much stronger structure than *V. parahaemolyticus* biofilm. SDW treatment had no obvious effect in biofilms. In contrast, AEW removed the biofilm cells and destroyed the high ordered structures, resulting in much less dense, and individually formed colonies when compared to the control (**Figures 7A-III,B-III**).

## Discussion

Biofilm formation of foodborne pathogens on food processing surfaces is a concern for the food industry (Wang et al., 2016). Controlling pathogen biofilm formation is hindered by EPS which limits the diffusion of sanitizers into the deepest layers of biofilms. In this study, we found that AEW had the ability to disrupt EPS and effectively eradicate foodborne biofilms.

We evaluated the effect of AEW on biofilm using a green fluorescent protein-tagged *E. coli* in preliminary experiments, which allowed non-destructive rapid microscopic visualization of biofilm population without using probes or dyes. There was a direct correlation between population and fluorescence signal of cells (Bron et al., 2006; Montañez-Izquierdo et al., 2012). Multilayers located at different depths and cells aggregates in the biofilms resulted in different fluorescence intensity (Webster et al., 2004). Hence, the eradication effect of AEW on biofilm could be clearly demonstrated with the direct observation of epifluorescence micrographs.

Acidic electrolyzed water electrolyzed at different NaCl concentration was characterized by pH, ACC, and ORP (**Table 1**). Different NaCl concentrations produced different ACC which affected the efficiency of AEW on *E. coli* biofilms (**Table 1**). High ACC causes changes in metabolic compounds within biofilm cells, causing cell death and removal. However, the pH and ORP of AEW were not the main factor contributing to bactericidal ability. A similar finding was reported by both Sun et al. (2012) and Vázquez-Sánchez et al. (2014) who concluded that the available chlorine in AEW might be one of the main factor for the inactivation of *S. aureus* biofilms. In addition, AEW inactivated bacteria due to the oxidative ability of ACC against the cell membrane, various metabolic functions, etc. (Huang et al., 2008). Specifically, AEW causes the degradation of bacterial protective barriers like EPS and increases membrane permeability. Other effects include the leakage of cellular inclusions, and decrease of activity of some key enzymes such as dehydrogenase (Zeng et al., 2010). Also, once the bacterial cells detach from EPS in the biofilm matrix, these cells are more vulnerable to sanitizer agents (Kumar and Anand, 1998).

In our study, the numbers of viable cells in the biofilms of *E. coli, V. parahaemolyticus*, and *L. monocytogenes* ranged from 6.77, 6.90, and 7.24 to 2.26, 3.33, and 1.34 log CFU/mL, respectively, after AEW-3 treatment for 5 min. *L. monocytogenes* biofilm was more susceptible to AEW compared to the other two Gram-negative pathogens. Similar results were obtained by Chen et al. (2015), who reported that the effectiveness of the sanitizer to *L. monocytogenes* was more remarkable than other Gram-negative bacteria. Moreover, our results were also supported by Skrivanova et al. (2006), who found that AEW was more effective against planktonic Gram-positive bacteria than planktonic Gram-negative bacteria. One explanation of these phenomena is that the transport of ions across the cell membrane of Gram-positive bacteria is more vulnerable to interference (Skrivanova et al., 2006; Feliciano et al., 2012; Chen et al., 2014). In addition, this apparent discrepancy could partly be explained by the different structural composition of EPS in different biofilms. There were great differences in the proportions of proteins and carbohydrates in EPS in biofilms formed by bacteria. Therefore, AEW efficiency in biofilm removal might vary according to the species of bacteria.

Extracellular polymeric substance makes up about 80% of the biofilm dry mass, primarily consisting of carbohydrates and proteins and plays a major role in mediating biofilms formation (Liu et al., 2007). EPS is responsible for biofilm properties, such as density, porosity, and hydrophobicity. An effective cleaning procedure should break up or dissolve the EPS in the biofilm so that disinfectants gains access to the viable cells (Shen et al., 2016). It is also reported that the main cause of biofilm removal is EPS degradation rather than removal of intracellular components (Christensen et al., 1990). For example, Gao et al. (2016) showed that the radical oxidants from CAT-NP activation of H_2_O_2_ degraded glucans in EPS via oxidative cleavage. Zhou et al. (2016) also observed that the presence of ozone reduced EPS contents to different extents. However, little is known whether AEW disrupts EPS thus resulting in biofilm eradication. Therefore, we attempted to determine the influence of AEW on biofilm characteristics systematically by investigating EPS content and composition (**Figure 3**). The experimental results show that AEW had a remarkable effect in the disruption of EPS in biofilms. Raman spectroscopic analysis combined with EPS chemical analysis revealed that the band intensities associated with carbohydrates, protein and nucleic acid were significantly decreased after AEW treatment when compared to the control treatment. The changes in the carbohydrate C-O-C group, tyrosine and phenylalanine of proteins were clearly observed. After exposure to AEW, the absence of 1,003 cm^-1^ and 1005 cm^-1^ bands in EPS were attributed to the ring deformation of phenylalanine and aromatic amino acids, indicating that denaturation or conformational changes caused cell death. It was in line with previous reports that the protein in EPS was decreased significantly by antibiotic agents (Jung et al., 2014).

This study shows that EPS disruption could be the basic mechanism of biofilm removal by AEW treatment. The present study hypothesized that the mechanism underlying EPS disruption and biofilms eradication upon exposure to AEW may be associated with ionic interactions. These interactions caused biofilm eradication by changing EPS hydrophobicity and localized charge along the polymer chains. Changes in charge and hydrophobicity would in turn affect the EPS structure. Future research could concentrate on the interaction between EPS and AEW. Some studies have also reported that there are differences in the degree of attachment and biofilm formation by the pathogen as affected by various types of food-contact surfaces, including stainless steel, glass, plastic, and wooden surfaces (Bang et al., 2014). Also, the efficacy of the sanitizer may be affected by surface type, which will be our next research step. In addition, the effect of AEW on mixed-species biofilm models akin to *in vivo* situation is certainly warranted.

Additionally, the residual cells which escapes after exposure to disinfection may further adhere and grow, resulting in a complex matrix (Molobela et al., 2010). These dispersed cells from biofilm showed stronger recalcitrance to disinfection than planktonic cells. Nevertheless, one finding from this study was that biofilm viable cells dispersed by AEW from the biofilm matrix into the ambient environment were under the detection limit, thus AEW treatment may not cause secondary pollution. Therefore, AEW is an excellent alternative to sanitizers and can be applied to control biofilms in food processing facilities as well as protecting foods from cross-contamination.

## Conclusion

In summary, this study indicated that AEW could effectively eradicate foodborne pathogen biofilms and not caused the secondary pollution. Therefore, AEW is a potent foodborne pathogen biofilms disrupter, which can be used as a reliable and eco-friendly alternative to sanitizer traditionally used in the food industry.

## Author Contributions

Conceived and supervised the study: YZ, YP, and HL. Designed the experiments: QH and XS. Performed the experiments: QH and XS. Analyzed the data: QH, JF, XS, ZZ, and XW. Revised the paper: ZZ, PM, and YZ. Wrote the paper: QH.

## Conflict of Interest Statement

The authors declare that the research was conducted in the absence of any commercial or financial relationships that could be construed as a potential conflict of interest.

## Footnotes

1 http://rsb.info.nih.gov/ij/
